# The CNS-specific proteoglycan, brevican, and its ADAMTS4-cleaved fragment show differential serological levels in Alzheimer’s disease, other types of dementia and non-demented controls: A cross-sectional study

**DOI:** 10.1371/journal.pone.0234632

**Published:** 2020-06-19

**Authors:** Ditte S. Jonesco, Morten A. Karsdal, Kim Henriksen

**Affiliations:** Biomarkers & Research, Nordic Bioscience, Herlev, Denmark; University of Florida, UNITED STATES

## Abstract

Evidence indicate that the brain-specific protein, brevican, is proteolytically cleaved during neurodegeneration, hence positioning fragments of brevican as potential blood biomarkers of neurodegenerative diseases, such as dementia. We aimed to develop two assays capable of detecting the brevican N-terminal (N-Brev) and the ADAMTS4-generated fragment (Brev-A), cleaved at Ser^401^, in serum and to perform a preliminary assessment of their diagnostic potential in dementias. Monoclonal antibodies against N-Brev and Brev-A were used to develop two ELISAs detecting each epitope. A comparison of brevican fragments in serum from individuals with AD (n = 28), other dementia (OD) (n = 41), and non-dementia-related memory complaints (NDCs) (n = 48) was conducted. Anti-N-Brev and anti-Brev-A antibodies selectively recognized their targets and dilution and spike recoveries were within limits of ±20%. Intra- and inter-assay CVs were below limits of 10% and 15%, respectively. For the N-Brev biomarker, serum from patients with OD showed significantly lower levels than those with AD (*p* = 0.05) and NDCs (*p* < 0.01). The opposite pattern was evident for Brev-A: serum levels in patients with OD were significantly higher than for AD (*p* = 0.04) and NDCs (*p* = 0.01). For both N-Brev and Brev-A, levels did not differ between AD and NDCs. The ratio of N-Brev/Brev-A resulted in increased significant differences between OD and AD (*p* < 0.01) and between OD and NDCs (*p* < 0.0001). The ratio discriminated between NDCs and OD (AUC: 0.75, 95% CI: 0.65–0.85, *p* < 0.0001) and between OD and AD (AUC: 0.72, 95% CI: 0.59–0.85, *p* < 0.01). In conclusion, we developed the first assays detecting the N-terminal of brevican as well as an ADAMTS4-cleaved fragment of brevican in blood. Differential levels of N-Brev and Brev-A between AD and OD allow for these biomarkers to possibly distinguish between different forms of dementias.

## Background

To date, several symptomatic treatments are available for AD but no disease-modifying therapy exist. Efforts to develop such therapies are in part hampered by an inability to diagnose patients early and accurately. Early and accurate diagnosis is difficult due to the initiation of AD pathology years before the first clinical manifestations [1,2] as well as the considerable overlap of clinical and pathological features of AD with other dementias such as dementia with Lewy bodies (DLB), vascular dementia (VaD) and fronto-temporal lobar dementia (FTLD) [3,4]. Diagnosing AD before clinical manifestations occur necessitates the existence of *in vivo* biomarkers reflecting pathophysiological changes in the brain. Current research diagnostic criteria are based solely on CSF and imaging biomarkers of amyloid and tau pathology [5]. Markers beyond amyloid and tau have also shown great potential. Neurogranin, a dendritic protein suggested to reflect synaptic degeneration, show increased CSF levels in early stage of AD, predict cognitive decline [6–8] and has been shown to differentiate between AD and other forms of dementia [9,10]. Although the CSF and neuroimaging markers of AD show high diagnostic accuracy, barriers exist in relation to their clinical use. Limiting factors include cost, invasiveness and accessibility of brain scans and lumbar punctures [11,12]. Considering these barriers, simple blood-measured biomarkers for diagnosis, progression of disease and for multiple sampling in clinical trials are attractive as they are low-cost with minimal complication risks and can easily be repeatedly assessed. Accordingly, huge efforts have been put into the development of blood-based biomarkers of AD [11,13]. The most successful blood-based biomarkers are those known from CSF [11,13]. Plasma biomarker profiles of Aβ and tau largely follow the pattern observed in CSF [14–16] although correlations of T-tau in plasma and CSF are not strong [16]. Another blood-based marker, Neurofilament light (Nfl) shows promise with diagnostic and prognostic abilities possibly even in preclinical stages of AD [17,18]. The application of biomarkers in both blood and CSF is challenged by the fact that the pathologies driving different types of dementia involve the same proteins, complicating the differential diagnosis. Thus, solely measuring protein levels is not likely to provide adequate information to distinguish between different types of dementias [19]. One possibility is to focus on enzyme-specific post-translational modifications (PTMs) of key proteins. This approach has already been utilized with markers like Aβ_1–42_ and P-tau. Targeting such neo-epitopes as biomarkers of dementia has gained much attention, as the protein fingerprint on key proteins is likely to provide insight into central disease mechanisms [20]. The strategy of targeting neo-epitope fragments in CSF and blood has provided several biomarkers with both diagnostic and prognostic potentials for AD [3,21–25]. Taken together, disease generated neo-epitopes measured in blood make good candidates for the ideal biomarkers of AD, defined as being non-invasive, simple to measure, inexpensive, reliable and able to reflect fundamental neuropathology [26].

Brevican is suggested to be an important component of highly organized extracellular matrix (ECM) structures named perineuronal nets (PNNs) [27]. These nets are lattice-like structures ensheathing the somatodendritic part of specific neuronal populations [28,29] and have been implicated in inhibition of synaptic plasticity [28,29] as well as biochemical protection of the neurons they ensheath [30–33]. Brevican is a CNS-specific chondroitin sulfate proteoglycan (CSPG) produced by astrocytes and neurons [34,35]. Several studies in mice show potential links between different types of memory and PNNs or more specifically, brevican as well as other CSPGs, in adult CNS [36–41]. In a mouse model of AD-like pathology, increased levels of brevican and other CSPGs were paralleled by impaired long-term potentiation (LTP) and contextual memory [42]. Interestingly, increased levels of the CSPGs occurred before the onset of Aβ deposition and LTP and contextual memory were restored by removal of CS by chondroitinase ABC (ChABC). This is in line with the suggested inhibitory function of PNNs. Also, a rat model of brain injury showed brevican to be upregulated in the area of the lesion as well as areas denervated by a lesion [43]. Furthermore, expression of brevican has been suggested to be increased in the superior frontal gyrus of AD patients compared to healthy controls [44]. In the CSF, increased levels of brevican were found to correlate with unfavourable outcome of patients with traumatic brain injury (TBI) [45]. In the same study, post-injury levels of brevican decreased with time to become significantly lower than levels observed in patients with neurodegenerative disease, indicating that a chronic disease state may also be reflected by increased CSF levels of brevican. However, investigations on CSF levels of brevican in AD patients found no differences between patients and controls [46].

Two isoforms of brevican exist. The major form is secreted into the extracellular space. The minor form, lacking the C-terminal part of the full protein, is attached to the cell membrane via a glycosylphosphatidylinositol (GPI)-anchor at Ala^646^ [35]. The >145 kDa protein is expressed with and without chondroitin sulfate side chains and contains a cleavage site at Ser^401^ (corresponding to Ser^396^ in rat) for a disintegrin and metalloproteinase with thrombospondin motifs (ADAMTS) family member [47,48]. ADAMTSs are expressed throughout the CNS with ADAMTS4 being the most abundant [49]. Rodent studies have shown induction of ADAMTS4 gene expression by CNS lesions and *in vitro* Aβ stimulation of astrocytes [50,51]. The ADAMTS4-cleaved brevican fragment is positively correlated to increase in ADAMTS4 gene expression as well as synaptic loss in the dentate gyrus [50,52]. Furthermore, expression of ADAMTS4-cleaved brevican was increased in the hippocampus of moderate to severe AD brains compared to healthy brains [53].

In summary, brevican is an important component of the PNNs suggested to be implicated in synaptic plasticity and neuroprotection. There is evidence of altered expression and cleavage of brevican in relation to AD and neurodegeneration in general. This places brevican and specifically the fragments resulting from ADAMTS4-cleavage as potential biomarkers of processes related to AD. Therefore, we set out to develop highly specific serum-based enzyme-linked immunosorbent assays (ELISAs) for detection of two distinct sites on brevican, the N-terminal (N-Brev) and the ADAMTS4-cleaved neo-epitope (Brev-A), with the potential to reflect pathological processes in the AD brain. We assessed the potential of these protein sites to provide information for differential diagnosis of dementia in a cross-sectional study encompassing non-demented subjects and patients diagnosed with AD or other forms of dementia.

## Material and methods

### Reagents and peptides

All chemicals were standard high-quality chemicals from Merck (Whitehouse Station, NJ, USA) and Sigma-Aldrich (St. Louis, MO, USA). Monoclonal antibodies (mAb) were produced and validated using synthetic peptides purchased from the Chinese Peptide Company (Beijing, China). Peptide conjugation reagents were produced by Pierce (Thermo Scientific, Waltham, MA, USA). For N-Brev, synthetic peptides include: a) Immunogenic peptide DVLEGDSSED-GGC-KLH (Keyhole-Limpet-Hemocyanin), b) screening peptide DVLEGDSSED-biotin, c) selection peptide DVLEGDSSED and d) elongated peptide **G**DVLEGDSSED. For Brev-A, synthetic peptides include: a) Immunogenic peptide SRGAIYSIPI-GGC-KLH (Keyhole-Limpet-Hemocyanin), b) screening peptide SRGAIYSIPI-biotin, c) selection peptide SRGAIYSIPI and d) elongated peptide **G**SRGAIYSIPI.

### Production of mAbs targeting Brev-A and N-Brev

The Anti-N-Brev antibody was raised against the decamer peptide ^23^DVLEGDSSED^32^ corresponding to the N-terminal sequence of mature brevican (present in both isoforms). The Anti-Brev-A antibody was raised against the decamer peptide ^401^SRGAIYSIPI^410^ corresponding to the neo-epitope sequence generated by ADAMTS4 cleavage of brevican at Ser401 (present in both isoforms). Both peptide epitopes were blasted for homology to other proteins using the NPS@:network protein sequence analysis with the UniProt/Swiss-Prot database [54].

The methods for producing these antibodies in mice were as previously described [55]. All the work on mice was approved by Beijing laboratory animal administration office and animal ethics committee of Nordic Bioscience (Beijing). Immortal antibody-producing hybridoma were produced as previously described [56]. Hybridoma cell lines specific to the selection peptide and without cross-reactivity to the elongated peptide in PBS buffer were selected for sub-cloning to ensure complete monoclonal hybridoma. Subsequently, monoclonal hybridomas were expanded to obtain a suitable volume of supernatant from which the antibodies were purified using a prepacked HiTrap^™^ Protein G Sepharose column (GE Healthcare Life Science, Buckinghamshire, UK). Furthermore, the anti-N-Brev antibody was labeled with horseradish peroxidase (HRP) using the Lightning link HRP labeling kit (Innova Bioscience cat. no. 701–0010, Cambridge, UK) according to manufacturer instructions.

### Development of ELISAs selectively detecting Brev-A and N-Brev

Using our monoclonal antibodies, competitive ELISAs were developed for selective detection of N-Brev and Brev-A. Preliminary experiments evaluating different buffers, incubation time and temperature as well as antibody and antigen concentrations, were performed to identify the optimal ELISA conditions. ELISA-plates used for the assay development were Streptavidin-coated (Roche cat. no.11940279). All ELISA plates were analyzed with the ELISA reader from Molecular Devices, SpectraMax M, (CA, USA).

The final settings for the N-Brev ELISA were as follows: A 96-well ELISA plate pre-coated with streptavidin was further coated with 1.4 ng/mL of the screening peptide for 30 min at 4°C, 300 rpm. The plate was washed five times in wash buffer (20 mM Tris, 0.1% Tween-20, 50 mM NaCl, pH 7.2). Hereafter, 20 μL/well of standard or sample was added followed by 100 μL/well of the peroxidase conjugated anti-N-Brev mAb diluted in assay buffer. The plate was incubated for 1 h at 4°C, 300 rpm and washed 5 times in wash buffer. Finally, 100 μL/well tetramethylbenzidine (TMB) (Kem-En-Tec cat. no. 4380) was added and the plate was incubated for 15 min at 20°C, 300 rpm. The colorimetric reaction was stopped by adding 100 μL/well of stopping solution (1% H_2_SO_4_) and the plate was measured at 450 nm with 650 nm as the reference. The screening peptide was diluted in coating buffer (8 mM Na_2_HPO_4_^.^12 H_2_O, 2 mM KH_2_PO_4_, 137 mM NaCl, 2.7 mM KCl, 0.1% Tween 20, 1% BSA, pH 7.4). Standards, samples and the anti-N-Brev mAb were diluted in assay buffer A (20 mM Na_2_HPO_4_^.^12 H_2_O, 4 mM KH_2_PO_4_, 34 mM NaCl, 2.7 mM KCl, 0.1% Tween 20, 1% BSA, pH 7.4).

The final settings for the Brev-A ELISA were as follows: A 96-well ELISA plate pre-coated with streptavidin was further coated with 1.5 ng/mL of the screening peptide for 30 min at 20°C, 300 rpm. The plate was washed five times in wash buffer. Hereafter, 20 μL/well of standard or sample was added followed by 100 μL/well of the anti-Brev-A mAb diluted in assay buffer. The plate was incubated for one h at 20°C, 300 rpm and washed five times in wash buffer. Then, 100 μL/well of peroxidase conjugated rabbit anti-mouse Ab (Jackson Immunoresearch cat. no. 315-035-045) was added and the plate incubated for one h at 20°C, 300 rpm followed by five times wash with wash buffer. Finally, 100 μL/well TMB was added to the wells and the plate was incubated for 15 min at 20°C, 300 rpm. The colorimetric reaction was stopped by adding 100 μL/well of stopping solution and the plate measured at 450 nm with 650 nm as the reference. The screening peptide was diluted in coating buffer. Standards, samples, anti-Brev-A mAb and peroxidase conjugated rabbit anti-mouse Ab were diluted in assay buffer B (40 mM Na_2_HPO_4_^.^12 H_2_O, 7 mM KH_2_PO_4_, 137 mM NaCl, 2.7 mM KCl, 0.1% Tween 20, 1% BSA, pH 7.4).

#### Standards

For the N-Brev serum assay, a standard curve was produced by 4-fold serial dilutions of the selection peptide in assay buffer A. Standard concentrations were: 0, 0.01, 0.05, 0.18, 0.73, 2.93, 11.72, 46.88, 187.50, 750, 3000 ng/mL.

For the Brev-A serum assay, a standard curve was produced by 4-fold serial dilutions of the selection peptide in assay buffer B. Standard concentrations were: 0, 0.05, 0.20, 0.78, 3.12, 12.50, 50, 200 ng/mL.

#### Technical validation

The assay range was characterized by the Lower Limit of Detection (LLOD) and the Upper Limit of Detection (ULOD). In this explorative setting, samples determined in duplicates, with CVs < 20% were accepted as reliable measurements if levels were above the LLOD, while levels below the LLOD were not considered reliable and thus given the value of the LLOD. The LLOD was established in an analytical run with 21 determinations of the lowest standard (i.e. assay buffer). The mean OD - 3x standard deviations (SD) equals LLOD. The ULOD was established based on 10 independent analytical runs determining the mean concentration of the highest possible standard concentration giving rise to an intra-assay CV < 10%. This mean concentration - 3x SD equals the ULOD.

The selectivity of the assays for their intended targets was assessed by performing an analytical run with serial dilutions of selection- and elongated peptides within the assay range. The degree to which a single amino acid (elongated peptide) prevented the binding of antibodies, represented the selectivity of the assays. Furthermore, the ability of the Brev-A ELISA to selectively detect ADAMTS4-cleaved rh-brevican, not full rh-brevican, was assessed as a supporting measure of selectivity.

Inter- and intra-assay variation was assessed by performing 10 independent analytical runs measuring duplicates of at least four different test samples consisting of serum as well as assay buffer spiked with selection peptide and calculating the mean CV%.

To assess potential differences in analyte detection between standard diluent and sample matrix, spiking recoveries were performed. For spiking recovery, two-four different serum samples were back-calculated from various amounts of spiked recombinant human brevican (rh-brevican) to estimate the recovery percentage. Linearity of dilution was assessed by performing dilution recoveries. Four different serum samples were back-calculated from 1:3, 1:4 and 1:5 dilutions to 1:2 diluted samples to estimate the recovery percentage. Serum samples were diluted in assay buffer as this was also the diluent for patient samples.

#### Cleavage of recombinant human brevican

*In vitro* cleavage of rh-brevican with rh-ADAMTS4 was performed by dissolving 15 μg of rh-brevican in cleavage buffer (50 mM Tris-HCL, 100 mM NaCl, 10 mM CaCl_2_, pH: 7.5) in a ratio of 1:2. 0.15μg of Rh-ADAMTS4 was added to the dissolved protein in a ratio of 1:100 and incubated at 37°C for 2, 24 and 72h. Cleavage was ended by placing the solutions at -80°C. Solutions were tested in the Brev-A ELISA.

### Immunohistochemistry

To investigate the spatial localisation of brevican, histology was performed on formalin fixed paraffin embedded (FFPE) human hippocampal tissue from a patient diagnosed post-mortem with Alzheimer’s Disease (BioChain cat. no. T2236052Alz). Tissue slides were incubated for 1h at 60°C, deparaffinized in toluene and rinsed in 99% ethanol. Endogenous peroxidase activity was blocked using hydrogen peroxide (0.45% H_2_O_2_ in 99% ethanol) for 30 min at RT. Then, tissues were sequentially hydrated in 96% and 70% ethanol and dH_2_O. To break formalin-induced methylene bridges, heat-mediated antigen retrieval was performed in citrate buffer (9.99 mM Tri-Sodium dehydrate, 0.05% Tween 20, pH 6) ON at 37°C. Slides were cooled to RT and washed in wash buffer before blocking with blocking buffer (0.5% casein in TBST) for 20 min. at RT. Tissues were incubated ON at 4°C with commercial available anti-brevican mAb (Merck Millipore cat no. MABN491), pre-neutralised anti-N-Brev mAb incubated ON at 4°C with selection peptide at a ratio of 1:50 moles) or non-neutralised anti-N-Brev mAb. Slides were then washed in wash buffer and incubated for 30 min at RT in HRP-labeled anti-mouse secondary antibody (Dako A/S, Glostrup, Denmark). Visualization was performed by incubation with 3,3’-diaminobenzidine (DAB) (Dako A/S, Glostrup, Denmark) for 10 min at RT and counterstaining was done using Mayer’s hematoxylin (3.3 mM Hematoxylin, 105.4 mM AlK(SO_4_)_2_, 12 H_2_O, 1 mM NaIO_3_) for 1–2 minutes until staining of nuclei was observed. Finally, the slides were sequentially dehydrated in H_2_O, 70%, 96%, 99% ethanol and toluene and visualized using an Olympus IX-70 microscope and Olympus DP71 camera.

### Western blotting

To investigate the CNS specificity of brevican, we performed sodium dodecyl sulfate-polyacrylamide gel electrophoresis (SDS-PAGE) and Western Blotting (WB) on 10 different tissues of rat: kidney, spleen, heart, liver, lung, muscle, hippocampus, frontal cortex, brainstem and cerebellum. First, protein extraction from snap frozen tissue was performed. Tissues were pulverised in a Bessman Pulveriser. Then, the powder was dissolved in extraction buffer (50 mM Tris-HCL, 50 mM HEPES, 5% glycerol, 1 mM EDTA, 0.5% C_24_H_39_NaO_4_, 1 protease inhibitor tablet from Roche for every 50mL buffer before use). To aid lysis, samples were treated with a homogenizer. After homogenization, samples were spun down at 4°C for 10 min at 10.000 g before collection of supernatant and storage at -80°C. Later, proteins in the lysates were separated on an SDS-PAGE gel under denaturing conditions. An equal amount of protein from each tissue was loaded on the gel by first determining the protein concentration of each sample, using a Bio-Rad protein assay kit (Bio-Rad), and then equalizing the protein concentrations with MilliQ water. Following separation on the gel, proteins were transferred to a membrane and the membrane was blocked in 5% milk-TBST for two h. Then, the membrane was incubated with anti-brevican mAb (Merck Millipore cat no. MABN491) and anti-p38 MAPK Ab (Cell Signaling) as a loading control ON at 4°C. Finally, membranes were washed in TBST and the primary antibodies were visualized using secondary peroxidase-conjugated mouse and rabbit antibodies followed by membrane development using ECL Western Blotting detection reagents (GE Healthcare).

### Biological evaluation of N-Brev and Brev-A

Using the developed ELISAs, levels of N-Brev and Brev-A were determined in accessible serum diluted 1:4 and 1:2, respectively, from patients diagnosed with AD (n = 28), Other Dementia (OD) (mainly VaD, LBD, FTLD and PDD) (n = 41) and patients with memory complaints originating from another causes than dementia (n = 48), (NDCs) (Table 1). All samples were derived from a previously described study [22]. Briefly, the study was carried out at the Department of Neurology, Lillebaelt Hospital, Vejle, Denmark between 2012 and 2014. The study group consisted of individuals with objective memory complaints referred to further investigation by other hospital departments and by general practitioners in the community. Patients underwent a diagnostic work-up of dementia consisting of clinical evaluation, lumbar puncture, neuroimaging (CT or MR) and cognitive assessment by mini mental state examination (MMSE) and neuropsychological evaluation based on Lurias Neuropsychological Investigation Methods. The clinical diagnosis was made according to the internationally standardized criteria [57,58]. CSF biomarkers were measured using commercially available kits from Fujirebio Europe N.V., Belgium: CSF Aβ1–42, (Art. no. 81576), t-Tau (Art. no. 81572), and p-Tau (Tau181P) (Art. no. 81574). After preparation, serum samples were stored at -80°C. The study cohort contains a group of 6 MCI subjects, which were not included in the analysis due to the low number. The study received approval from the Danish Ethics Committee for the Capital Region of Denmark in accordance with the Helsinki declaration and written informed consent was given by all participants.

**Table 1:** Cohort characteristics.

	*AD*	*OD^#^*	*NDCs*	*Differences*
No. of patients	28	41	48	-
Male/Female	18/10	19/22	21/27	ns
Age	73(8.8)	69(9.6)	61(11.0)	*
BMI	23(4.5)	25(4.5)	26(4.7)	ns
No. MMSE scored	13	15	12	-
MMSE	22(3.8)	23(4.1)	28(2.7)	*
CSF Aβ	340.5(258–406)	468.0(351–758)	713.0(579–875)	***
CSF T-tau	341.0(219–512)	259.0(166–347)	221.0(155–279)	**
CSF P-tau	74.5(55–86)	55.0(32–72)	52.0(34–65)	**

Demographics and diagnostic evaluation is indicated for each group. Age, BMI and MMSE score are presented as mean(SD). CSF levels of Aβ, T-tau and P-tau are presented as median(IQR). The number of participants with an MMSE score in each diagnostic group is indicated. ‘*’ denotes that the characteristic is significantly different in AD and OD compared to NDCs. ‘**’ denotes that the characteristic is significantly different between AD and NDCs. ‘***’ denotes that the characteristic is significantly different between all groups. ^#^ ‘Other dementia’ include the following conditions (no.): vascular dementia (10), fronto-temporal lobar dementia (6), dementia with lewy bodies (4), parkinson’s disease (3), Normal Pressure Hydrocephalus (2), depressive pseudo-dementia (2), apoplexia (2), aphasia (2), paraneoplasia (1), epilepsy (1), hippocampal atrophy (1), pick’s disease (1), not specified (6).

### Statistical analysis

Differences between groups were tested using the *Kruskal–Wallis test* by ranks. Dunn’s pairwise tests were carried out for the three pairs of groups. Results are shown as Scatter plots with the median and 95% CI indicated. Correlation analysis were performed using the Spearman’s Rank correlation Coefficient. The significance level was set at 0.05. Graphs and statistical analyses were performed using GraphPad Prism version 7 (GraphPad Software, Inc., CA, USA) and MedCalc Statistical Software version 14.8.1 (MedCalc Software bvba, Ostend, Belgium; http://www.medcalc.org; 2014).

## Results

### Characterisation of N-Brev and Brev-A ELISAs

#### Selectivity of the ELISAs

**Fig 1:**
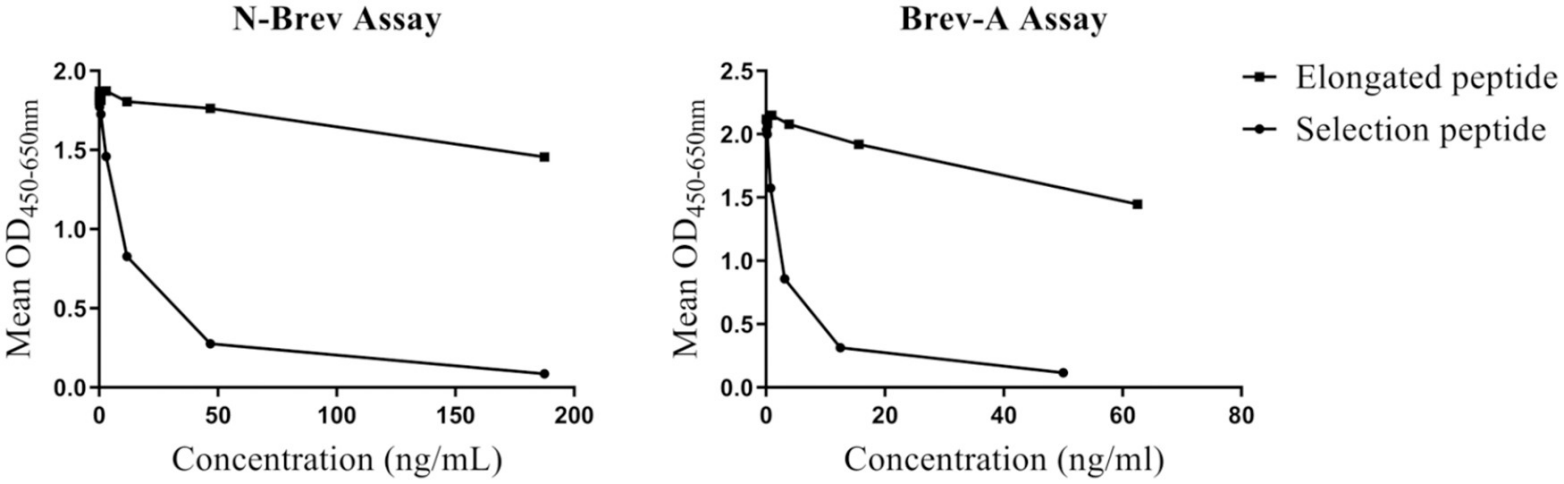
Peptide selectivity test of monoclonal anti-N-Brev and anti-Brev-A antibodies. The binding of antibodies to selection peptides and elongated peptides of concentrations within the measuring range of each assay are shown. For both antibodies, an addition of a single amino acid (elongated peptide) disrupted the binding to a satisfying extent. Selection and elongated peptides were diluted in assay buffer.

**Fig 2:**
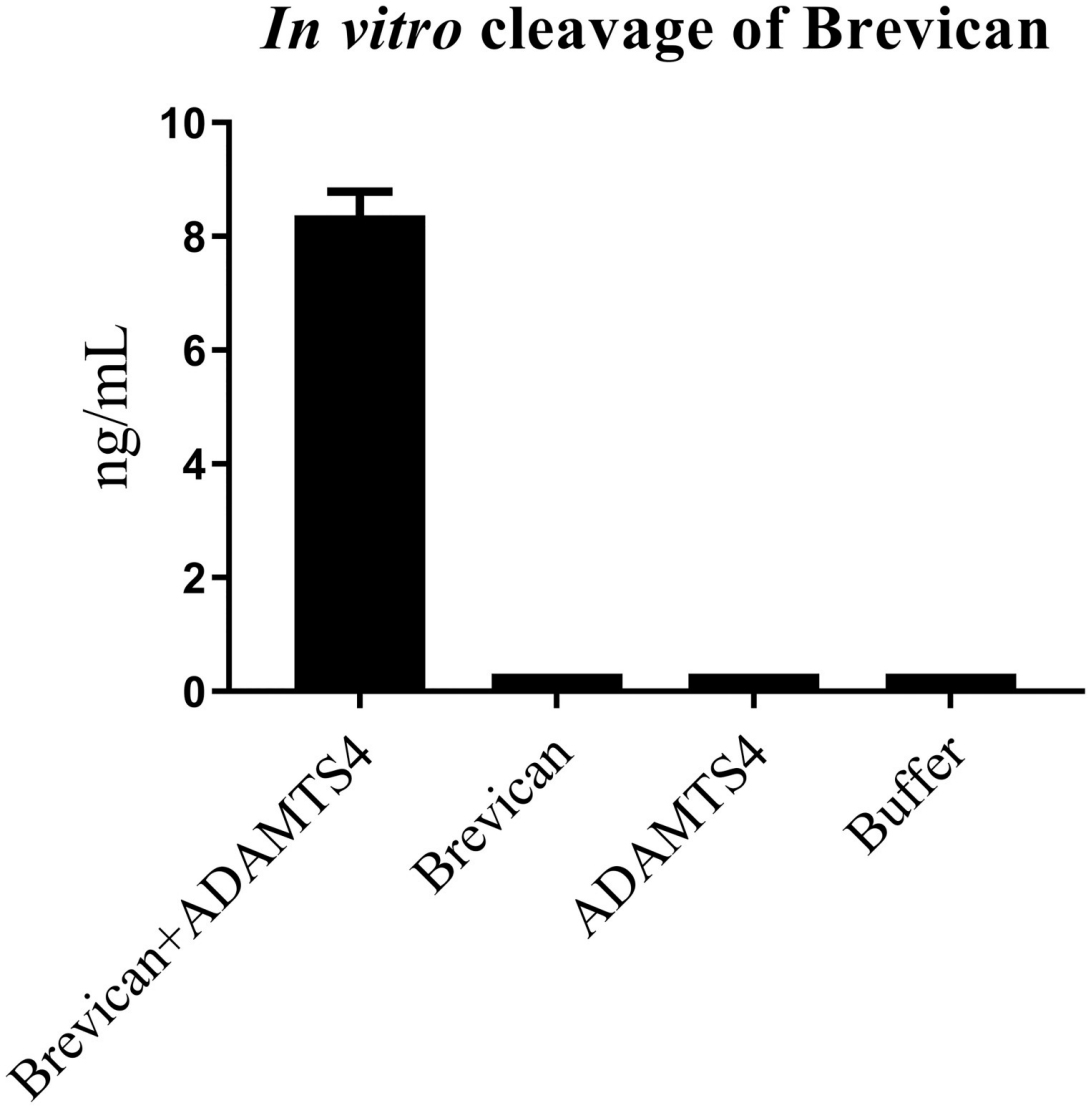
Selectivity test of Brev-A ELISA. The signal obtained by Brev-A ELISA in response to ADAMTS4-cleaved brevican, non-cleaved brevican, ADAMTS4 and cleavage buffer incubated for 72 h are shown. Concentrations below LLOD was given the value of LLOD. Shown are the mean of two determinations plotted with the standard deviation.

The anti-N-Brev and anti-Brev-A monoclonal antibodies showed high selectivity for their 10 amino acid epitopes, ^23^DVLEGDSSED^32^ and ^401^SRGAIYSIPI^410^, respectively, as the addition of a single amino acid at the N-terminal of both epitopes reduced the binding of antibody almost completely (Fig 1). Blasting the epitope sequences of N-Brev and Brev-A revealed no homology to other proteins than brevican and the sequences were found in both isoforms of the protein (S1 and S2 Figs). In order to characterize the selectivity of the Brev-A ELISA further, we investigated the ability of the ELISA to detect *in vitro* cleavage products of rh-brevican by ADAMTS4. Only ADAMTS4 cleaved brevican, not full length brevican, ADAMTS4 or cleavage buffer, was detected in the ELISA (Fig 2).

#### Technical validation

The measuring ranges of the N-Brev and Brev-A assays were defined as the range of analyte concentration from LLOD to ULOD determined to be 0.9–131.3 ng/mL and 0.3–45.3 ng/mL, respectively. To determine the optimal dilution of human serum samples, the effect of serum matrix on our antibodies was assessed by calculation of the dilution recovery. The Brev-A assay displayed a good linearity with analyte dilution recovery percentages within 100±20% throughout the dilution range. For the N-Brev assay, good linearity with analyte dilution recovery percentages within 100±20% was obtained only in human serum with a dilution of minimum 1:4 (S1 Table). Possible differences in analyte detection between standard diluent and sample matrix of N-Brev and Brev-A assays were investigated by calculation of the spiking recoveries of full-length and ADAMTS4-cleaved rh-brevican, respectively. Both assays displayed no discrepancies reflected by mean spiking recoveries of 99% and 105% for N-Brev and Brev-A, respectively (S2 Table). Both assays displayed high technical stability as inter- and intra-assay variation of N-Brev assay was 10.0% and 3.7%, respectively and inter- and intra-assay variation of Brev-A assay was 7.9% and 5.3%, respectively. Data of the technical validations are summarised in Table 2.

**Table 2:** Technical specifications of N-Brev and Brev-A ELISAs.

	*N-Brev*	*Brev-A*
Detection range (LLOD—ULOD)	0.9–131.3 ng/mL	0.3–45.3 ng/mL
Intra-assay variation	3.7%	5.3%
Inter-assay variation	10.0%	7.9%
Dilution recovery	116%	110%
Spiking recovery	99%	105%

Technical parameters of N-Brev and Brev-A ELISA are listed. Dilution recovery percentages are reported for the optimal dilution of each assay.

### Investigating the CNS specificity of brevican

**Fig 3:**
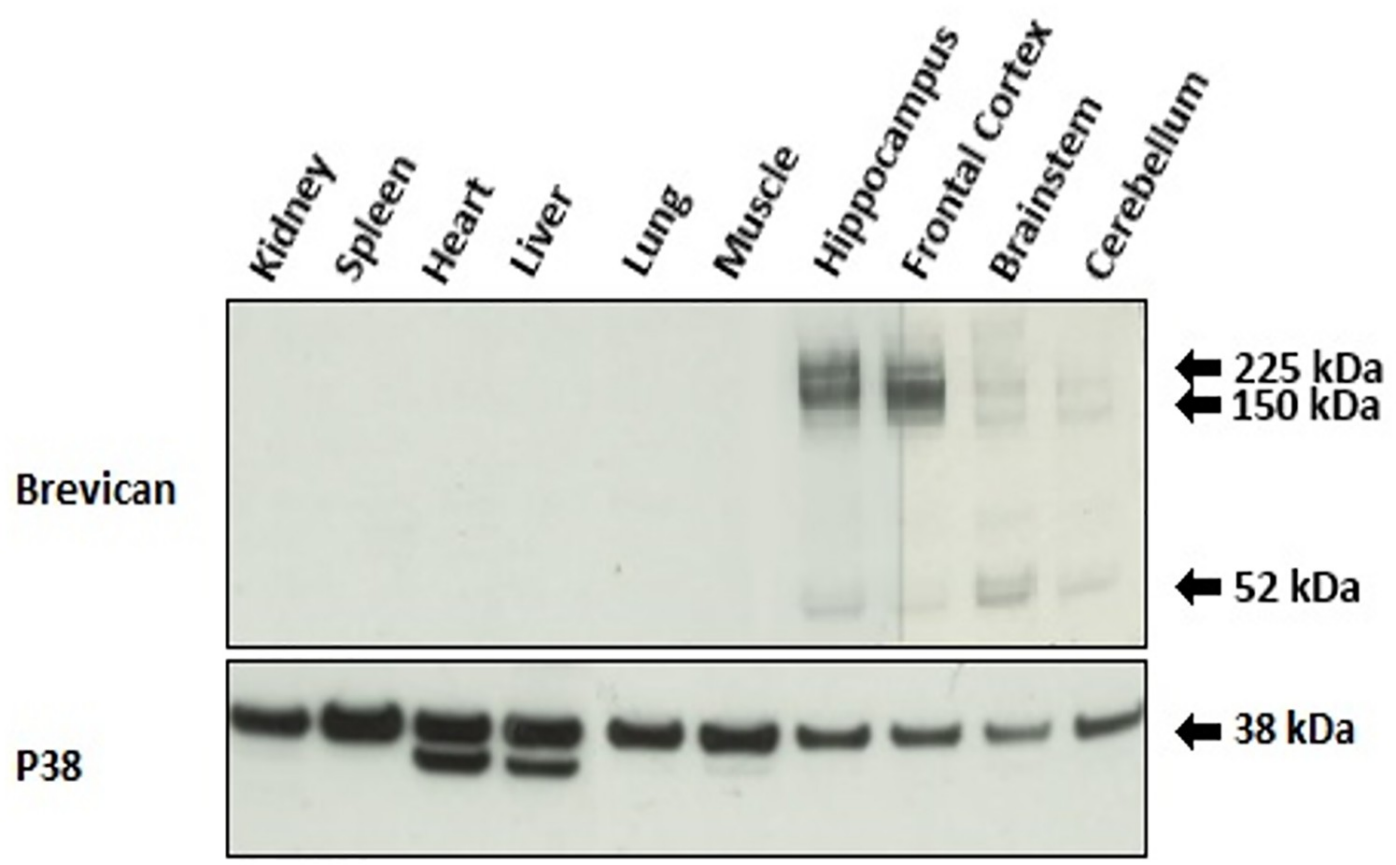
Specific localization of brevican to tissues of the CNS. A Western blot performed on homogenates of 10 different rat tissues is shown (n = 1). A commercial antibody targeting brevican visualizes protein bands around 150 to 250 kDa and a faint band around 50 kDa only in tissue of the rat CNS. The original and unadjusted images underlying this figure are presented in Supporting Information.

The presence of brevican in kidney, spleen, heart, liver, lung, muscle, hippocampus, frontal cortex, brainstem and cerebellum of rat was investigated using tissue extraction and visualization of brevican on WB using a commercial anti-brevican antibody. Anti-brevican antibodies detected bands solely in tissues of the brain (Fig 3). Further, the bands of brevican were most pronounced in tissue of the hippocampus and frontal cortex.

### Spatial localization of brevican in hippocampal tissues of the AD brain

**Fig 4:**
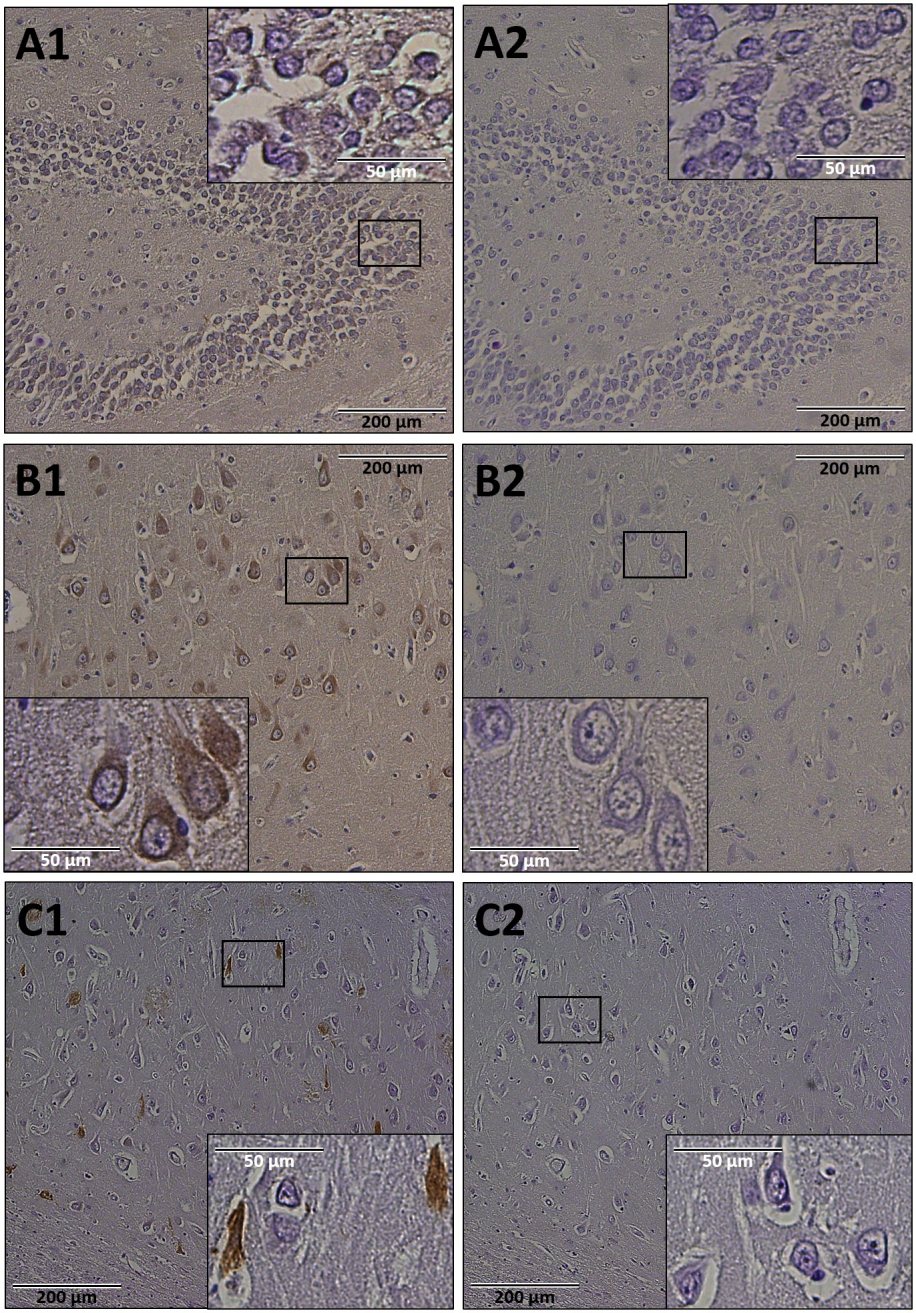
Spatial localization of brevican in human hippocampal AD brain tissue. Shown are two different locations in human hippocampal AD brain tissue incubated with anti-N-Brev antibodies (A1, B1); anti-N-Brev antibodies pre-incubated over night with N-Brev selection peptides in a ratio of 1:50 moles (A2, B2) and commercial anti-brevican antibodies (C1) with a blank control (C2). The extracellular matrix and the cytoplasm of neurons are stained. (n = 1).

Anti-N-Brev antibodies stained the ECM and the cytoplasm of neurons in two different areas of the hippocampus (Fig 4A1 and 4B1). mAbs pre-incubated with N-Brev selection peptide included as a control did not produce a signal (Fig 4A2 and 4B2). A commercial mAb against brevican primarily stained the neuronal cytoplasm (Fig 4C1). Differences in staining patterns of N-Brev and commercial brevican antibodies possibly reflect differences in brevican epitopes targeted by these antibodies.

### Serum N-Brev and Brev-A levels in AD, OD and NDCs

**Fig 5:**
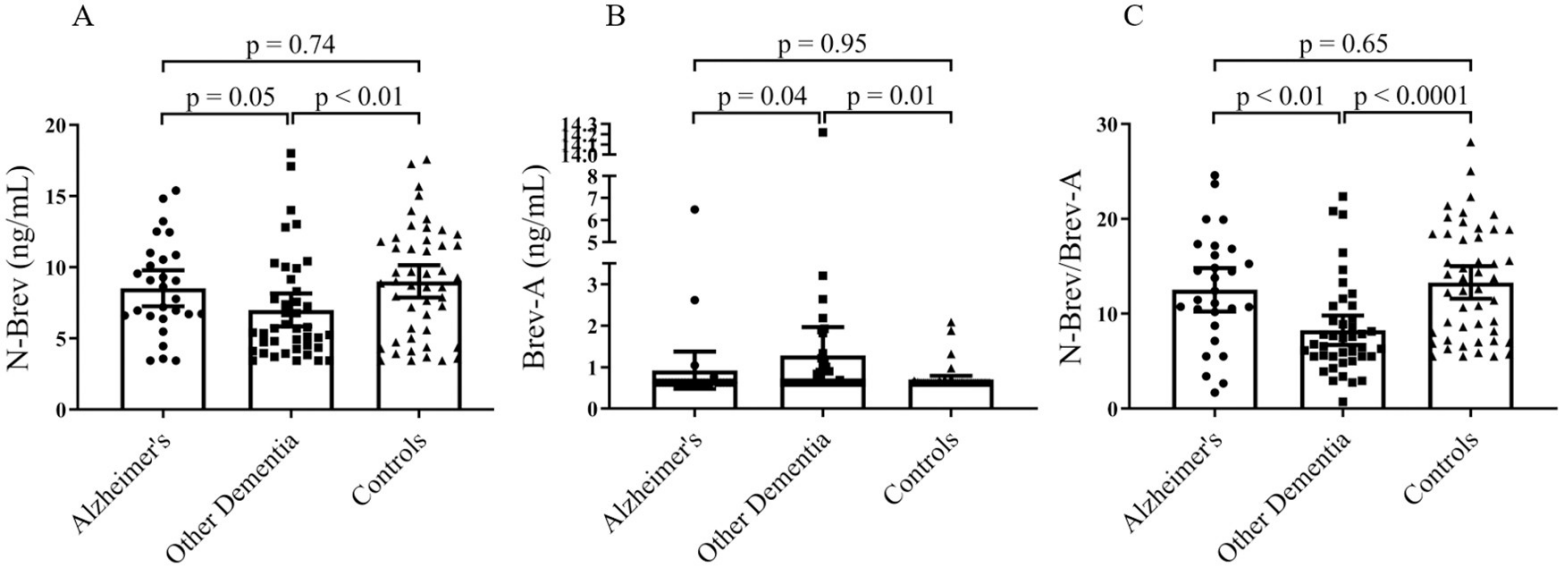
Detection of N-Brev and Brev-A in serum from NDCs and patients diagnosed with AD or ODs. Levels (Mean with 95% CI) of N-Brev (A) and Brev-A (B) measured in N-Brev and Brev-A assays in serum of NDCs and patients diagnosed with AD or ODs are depicted in scatter dot plots. In (C), ratios of N-Brev and Brev-A are depicted. Significance of differences in biomarker levels is determined using Kruskal-Wallis H Test of Mean Ranks adjusted for multiple testing.

A summary of cohort characteristics is given in Table 1. Briefly, patient demographics were comparable between groups with the exception of mean age being significantly different in NDCs compared to both AD and OD. As expected, mean MMSE score and core AD CSF biomarker levels differed between groups. Both N-Brev and Brev-A were detectable in serum of patients with AD and OD as well as NDCs. N-Brev was detected at levels above LLOD in 93% of serum samples. Brev-A was detected at levels above LLOD in only 24% of samples. For the N-Brev biomarker, serum from patients with OD showed significantly lower levels compared to that of AD (*p* = 0.05) and NDCs (*p* < 0.01) with mean ranks of 47.5, 63.5 and 66.2, respectively (Fig 5). The opposite was seen for Brev-A; serum levels in patients with OD were significantly higher compared to AD (*p* = 0.04) and NDCs (*p* = 0.01) with mean ranks of 67.9, 54.5 and 54.1, respectively. For both markers, levels did not differ between AD and NDCs (*p* = 0.74 for N-Brev and *p* = 0.95 for Brev-A). Looking into the ability of serum brevican to reflect pathophysiology related to AD, we performed correlation analyses on serological levels of N-Brev and CSF levels of core biomarkers of AD. Brev-A levels were not investigated here, as the large percentage of samples with the value of LLOD would prevent reliable interpretation of the correlation matrix. Levels of brevican showed modest correlation with CSF P-Tau (S3 Table). There was no correlation between N-Brev and Aβ or T-tau.

Given the variation in serum levels of N-Brev in all subjects of this cohort, we speculated that, besides the possibility of disease-related alterations in total brevican expression, basal expression levels of total brevican may vary between individuals. In this case, normalisation via ratios could allow for a more detailed picture of pathology possibly reflected by ADAMTS4 cleavage of brevican. Ratios of N-Brev/Brev-A serum levels were significantly lower in OD compared to both AD (*p* < 0.01) and NDCs (*p* < 0.0001) with mean ranks of 40.8, 66.5 and 70.2, respectively. No difference in ratios was observed between AD and NDCs (*p* = 0.65).

**Fig 6:**
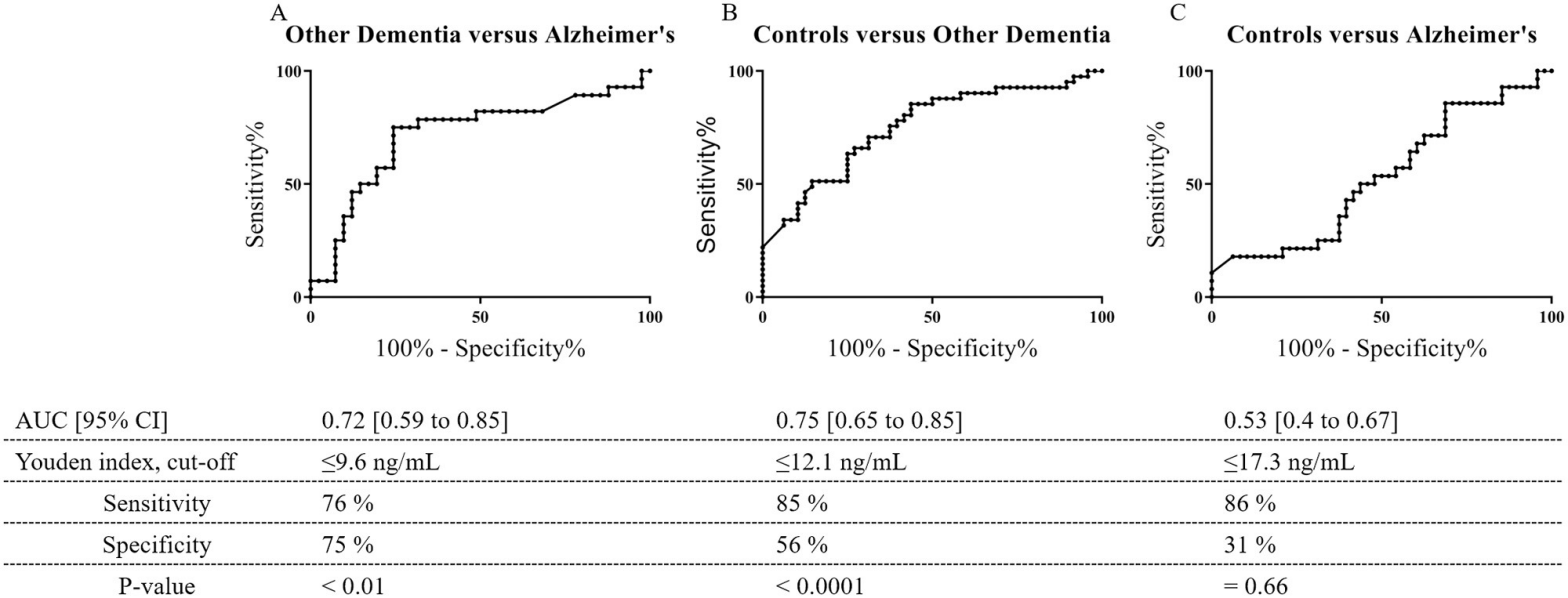
ROC curve for discrimination between dementia groups and controls. The results of ROC curve analysis for the ratio of serum levels of N-Brev and Brev-A are depicted. The table shows the AUC; optimal cut-off values (calculated by the Youden index); and the corresponding measures of sensitivity and specificity. The graph show (A) OD versus AD, (B) NDCs versus OD, (C) NDCs versus AD.

ROC analysis was performed to further access the ability of the N-Brev/Brev-A ratio to differentiate between the different groups of the cohort. N-Brev/Brev-A were significantly better than chance at discriminating between NDCs and OD (AUC: 0.75, 95% CI: 0.65–0.85, *p* < 0.0001) as well as OD and AD (AUC: 0.72, 95% CI: 0.59–0.85, *p* < 0.01). No separation was obtained between AD and NDCs (AUC: 0.53, 95% CI: 0.40 to 0.67, p = 0.66) (Fig 6). Optimal cut-off values of the N-Brev/Brev-A ratio, defined by the Youden index, allowed for separation of AD and OD from NDCs with sensitivities and specificities of 86% and 31% as well as 85% and 56%, respectively (Fig 6). AD and OD were separated with a sensitivity of 76% and a specificity of 75%. ROC analyses were also performed on each marker separately. N-Brev levels separated OD from AD and NDCs with AUC of 0.66 (p = 0.03) and 0.65 (p = 0.02), respectively. Likewise, Brev-A levels separated OD from AD and NDCs with AUC of 0.61 (p = 0.04) and 0.62 (p = 0.01), respectively. On the other hand, N-Brev and Brev-A levels did not separate AD from NDCs (AUC: 0.54 and 0.50, p > 0.05, respectively). Results of ROC curve analysis for each marker alone is presented as supplementary data (S4 Table).

## Discussion

In this study, we present two competitive ELISAs detecting distinct sites on the CNS-specific ECM protein, brevican. To our knowledge, there are no commercially available assays specifically detecting these distinct sites, thus highlighting the novelty of the data presented here. These sites are quantified in serum of AD, OD and NDCs using our competitive ELISAs, suggesting that 1) brevican and/or fragments of brevican are transferred from the CNS into the peripheral blood compartment and 2) our assays are capable of quantifying the level of these potential markers. Overall, this allows the possibility that brevican has potential as a blood-based biomarker of CNS disease and, given that the sensitivity of measurements is improved, our assays have potential for clinical utility.

To build the assays, we raised mAbs selectively recognizing the N-terminal of mature brevican, N-Brev, or the neo-epitope on brevican-fragments generated by ADAMTS4-cleavage at Ser^401^, Brev-A. The in-house ELISAs showed low serum matrix interference as well as high technical reliability with inter- and intra-assay variations well below the acceptable limits. Both ELISAs showed little cross-reactvity towards an elongated peptide suggesting high selectivity towards the intended target. Selectivity of the Brev-A assay was further supported by specific detection of *in vitro* ADAMTS4-cleaved rh-brevican and not full-length rh-brevican which, unlike the screening and elongated peptides, displayed a natural tertiary structure better resembling a true biological condition. We note that the N-Brev assay also detected rh-brevican as evident by the spiking recovery tests (S2 Table). Overall, the analysis performed to assess the reliability of measurements by the ELISAs show a good performance of both assays.

Staining performed with the in-house anti-N-Brev antibody and a commercial anti-brevican antibody suggests cytoplasmic localisation of brevican in hippocampal neurons of the AD brain. In line with these observations, brevican has previously been visualized inside neurons of the human hippocampus [53] and the protein is present in the microsomal membrane fraction of rat brain membrane preparations [35]. In addition, the N-Brev antibody also stained the hippocampal ECM. This finding is in agreement with previous reports on the presence of brevican in brain ECM [28,29,53,59]. Brevican is known to be posttranslationally processed [52,53,60]. Differences in staining patterns of N-Brev and commercial brevican antibodies possibly reflect distinct populations of processed brevican protein targeted by these antibodies.

Running homogenates of different rat tissues on a WB and staining with a commercial anti-brevican antibody revealed bands exclusively in the CNS tissue. The bands corresponded to previous experimentally determined molecular weight of brevican [47,48,50,52]. This finding supports the current knowledge of brevican being a CNS-specific protein. Importantly, we were able to quantify both N-Brev and Brev-A in serum using our assays.

We observed significant differences in serum levels of both markers between AD and OD while no separation was observed between AD and NDCs. Furthermore, both markers separated OD from NDCs. The few published studies investigating the level of brevican and its proteolytic fragments in normal versus neurodegenerative conditions suggest that an increase of total level of brevican and ADAMTS4-cleaved brevican is associated with neurodegeneration [42–45,50,52,53]. In this study, Brev-A serum levels were increased in OD compared to NDCs. This finding is in line with previously published studies on the subject [50,52,53] although these studies were performed only in brain tissue, either homogenized and visualized on a WB or visualized on tissue slides using methods of immunohistochemistry. For N-Brev, serum levels were decreased in OD compared to NDCs. This finding contradicts observations made by others in brain tissue from both animal models of neurodegeneration [42,59] and AD patients [44] as well as in CSF from AD patients [46]. Again, results of these studies are not directly comparable to our study in blood. We performed correlation analyses in order to examine the ability of serum brevican to reflect pathophysiology related to dementia. We observed a modest correlation between serological levels of N-Brev and CSF levels of P-tau possibly reflecting a link between levels of N-Brev and tau pathology.

Interestingly, combining our markers into ratios increased the observed differences between groups. Given the variation in serum levels of N-Brev in all subjects of this cohort, it is tempting to speculate that, besides the possibility of disease-related alterations in total brevican expression, normal expression levels of total brevican may vary between individuals, in which case normalisation via ratios could allow for a more detailed picture of pathology possibly reflected by ADAMTS4 cleavage of brevican as suggested in the literature [50,52,53]. This rationale is similar to the rationale applied as a possible explanation for the improved performance of the Ab42/Ab40 ratio over Aβ42 alone [61]. ROC curve analysis, showing that the biomarker ratio can significantly differentiate between OD and AD as well as OD and NDCs, gives a positive indication that these markers might be applied in connection with other methods for differentiation between healthy individuals and different types of dementia, although these data are to be interpreted with caution due to the relatively small cohort applied in this study.

Using this cross-sectional cohort of dementia, we have performed investigations on the diagnostic potential of N-Brev and Brev-A. The design of the cohort does not provide insight into the markers’ potential for prognosis or ability to monitor efficacy of treatment. Importantly, lack of diagnostic power does not necessarily exclude prognostic power and thus, investigations on the potential for prognosis and monitoring efficacy of treatment of both N-Brev and Brev-A are warranted. Furthermore, levels of total and fragmented brevican may be of interest in other more traumatic forms of neurodegeneration, like traumatic brain injury (TBI), as alteration in brevican levels, as well as modification of brevican, has been shown in brain lesion models [43,50,52] as well as in patients with TBI [45].

There are obvious advantages of the use of blood biomarkers. However, the CNS is separated from the peripheral blood by the blood-brain barrier (BBB) possibly affecting the peripheral composition of CNS-derived protein species. Therefore, an interesting next step in our investigation on the biology underlying the release of N-Brev and Brev-A from the brain parenchyma is the measurement of these markers in CSF. In the present study, we did not have access to patient CSF.

The presence of the BBB raises the concern that only small fractions of brain proteins enter the blood. Indeed, this study shows that sensitivity of measurements is a major challenge when targeting CNS-specific proteins in blood. One limitation of this study was that only 24% of subjects had Brev-A levels higher than the LLOD. The remaining subjects were given the value of the LLOD. Biologically, it makes sense that the pool of brevican fragments cleaved by a specific protease at a specific site (Brev-A) would amount to less than the pool of full-length brevican including all possible fragments of brevican (N-Brev). However, from a technical point of view, the low concentration of Brev-A in serum necessitates highly sensitivity detection methods in order to separate low Brev-A values of one subject from that of another. Correct quantification would provide a more detailed picture of Brev-A levels and possibly improve separation of different subject groups. From this study it is clear that sensitivity of the assay needs to be improved going forward.

Another limitation of this study was the heterogeneity of the group “other dementia.” The heterogeneity makes it difficult to set forth solid conclusions based on the observed differences in biomarker-levels between this group, AD and NDCs. It is possible that the variation of N-Brev and Brev-A within the group of OD merely reflects the heterogeneity of pathology. On the other hand, an equivalent variation in biomarker-level is observed in the two other groups. The difference in mean age between all demented and NDCs is also a limitation of this cohort as we cannot exclude the effect of age on the level of biomarkers.

In summary, we developed the first assays detecting the N-terminal of brevican as well as an ADAMTS4-cleaved fragment of this protein in blood. In rat, we showed specific expression of brevican in the CNS and this finding is in line with the scientific consensus on brevican as a protein of the CNS. The most important and straightforward finding of this study is the detection and quantification of this CNS protein in blood of both demented and non-demented subjects. This finding allows for the possibility of gaining information on the pathology underlying CNS diseases in a non-invasive and inexpensive manner.

Investigations on the diagnostic potential of N-Brev and Brev-A are positive as levels of both markers showed differential expression between groups. However, it is not clear from this study whether our markers reflect an AD-specific profile. Investigations on the potential of N-Brev and Brev-A within prognosis and monitoring of efficacy of treatment is of great interest as is the case for the potential of the markers within other types of neurodegeneration.

## Supporting information

S1 Fig Significant alignments of the epitope sequence of N-Brev. Shown are the three sequences producing significant alignments to the epitope sequence of N-Brev. The sequence was blasted for homology to other proteins using the “NPS@: Network Protein Sequence Analysis with the UniprotKB/Swiss-prot database” software online.
(TIF)

S2 Fig Significant alignments of the neo-epitope sequence of Brev-A. Shown are the five sequences producing significant alignments to the neo-epitope sequence of Brev-A. The sequence was blasted for homology to other proteins using the “NPS@: Network Protein Sequence Analysis with the UniprotKB/Swiss-prot database” software online.
(TIF)

S1 Table Dilution recovery of N-Brev and Brev-A ELISA. Going left to right, the columns contain information on: the extent of dilution, concentrations of N-Brev or Brev-A in diluted serum samples, percent recovery of N-Brev or Brev-A in serum diluted from 1:2 to 1:5. Optimal dilution for each assay is indicated by black square.
(DOCX)

S2 Table Spiking recovery of N-Brev and Brev-A ELISA. Going left to right, the columns contain information on: measured concentrations of N-Brev and Brev-A in serum samples, measured concentration of cleaved or full length rh-brevican for spiking, expected concentration of spiked serum samples, measured concentration of spiked serum samples, percent recovery of cleaved or full length rh-brevican and mean percent recovery in all samples.
(DOCX)

S3 Table Correlation between N-Brev and core CSF biomarkers of AD. Listed are the Spearman’s rho correlation coefficients (r) with the 95% confidence interval. Aβ, amyloid-β; T-tau, total tau; P-tau, phosphorylated tau; n, number of patients.
(DOCX)

S4 Table ROC curve for discrimination between dementia groups and controls. For ROC curve analysis performed on serum levels of N-Brev and Brev-A, reported are the AUC, optimal cut-off values calculated by the Youden index and the corresponding measures of sensitivity and specificity for each comparison.
(DOCX)

S1 Raw images (TIF)
