# Supplementary figures and images for: The CNS-specific proteoglycan, brevican, and its ADAMTS4-cleaved fragment show differential serological levels in Alzheimer’s disease, other types of dementia and non-demented controls: A cross-sectional study
Source: PLoS One. 2020 Jun 19;15(6):e0234632. doi: 10.1371/journal.pone.0234632 (PMC7304580; doi:10.1371/journal.pone.0234632)

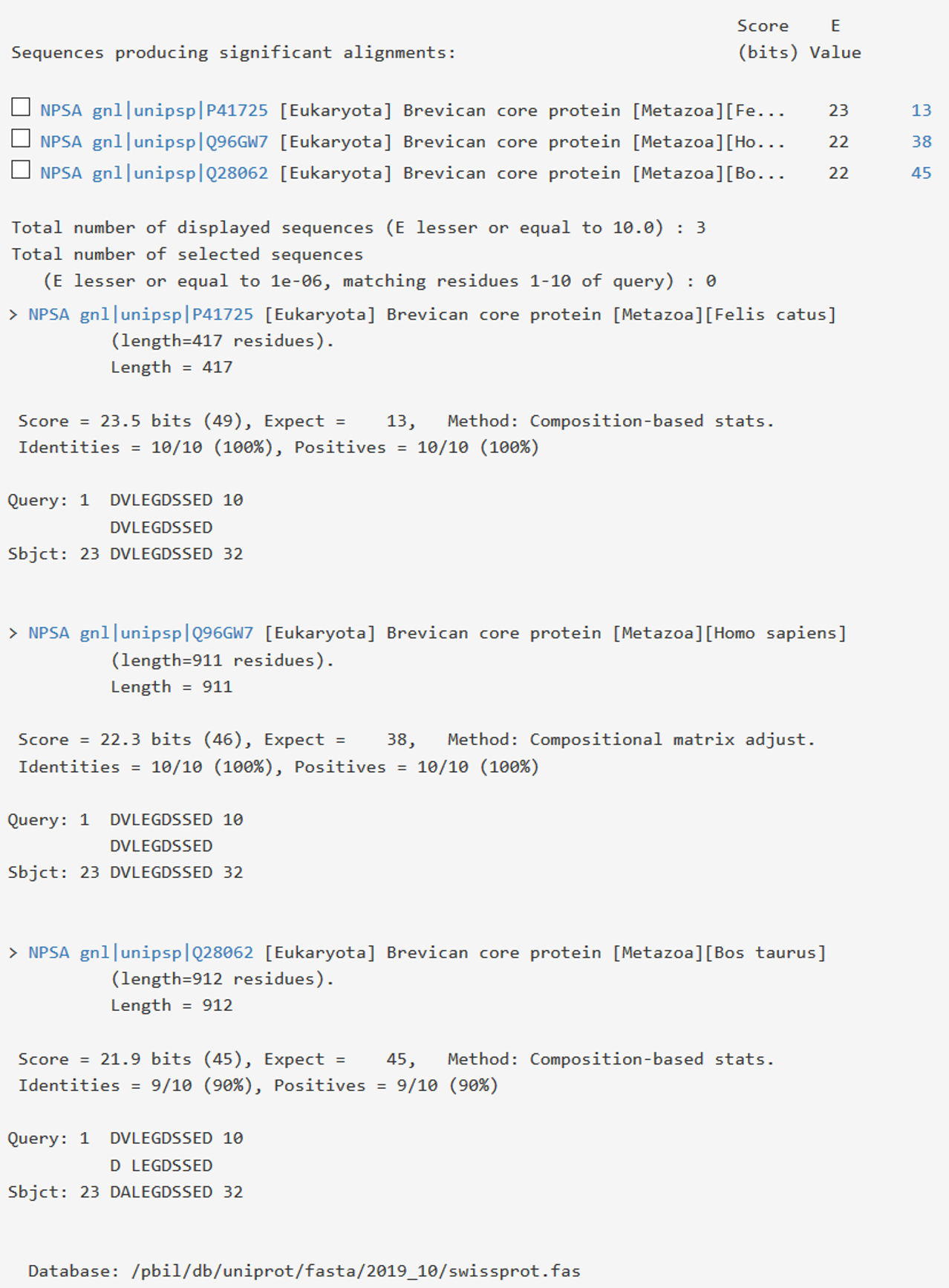

Supplement: S1 Fig — Shown are the three sequences producing significant alignments to the epitope sequence of N-Brev. The sequence was blasted for homology to other proteins using the “NPS@: Network Protein Sequence Analysis with the UniprotKB/Swiss-prot database” software online. (TIF) [file pone.0234632.s001.tif]

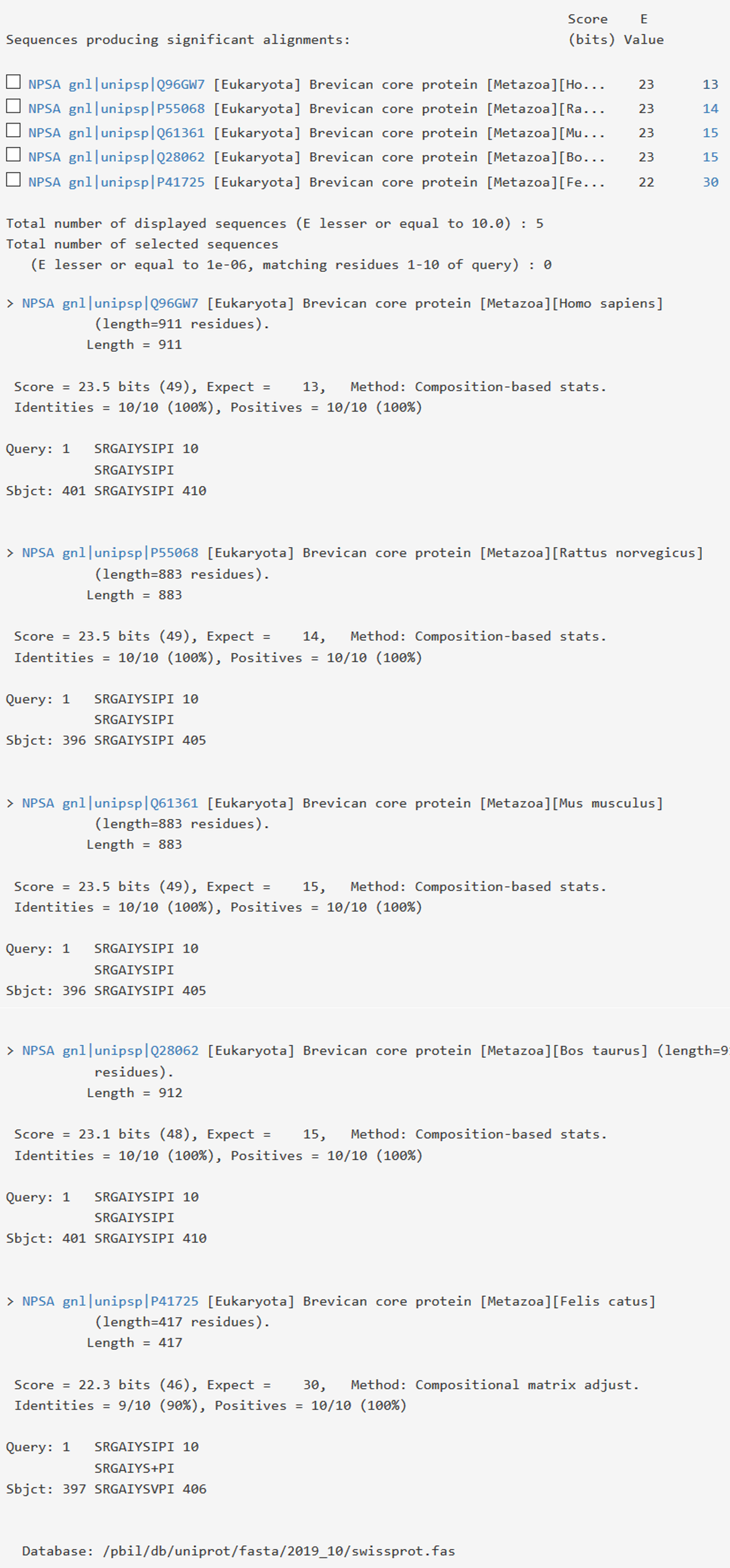

Supplement: S2 Fig — Shown are the five sequences producing significant alignments to the neo-epitope sequence of Brev-A. The sequence was blasted for homology to other proteins using the “NPS@: Network Protein Sequence Analysis with the UniprotKB/Swiss-prot database” software online. (TIF) [file pone.0234632.s002.tif]

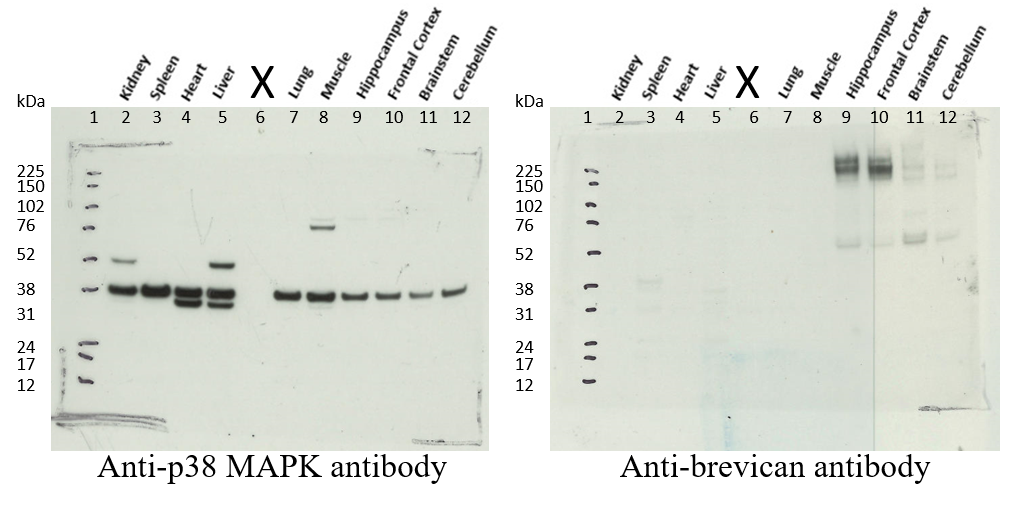

Supplement: S1 Raw images — (TIF) [file pone.0234632.s007.tif]
