# Supplementary material for: The CNS-specific proteoglycan, brevican, and its ADAMTS4-cleaved fragment show differential serological levels in Alzheimer’s disease, other types of dementia and non-demented controls: A cross-sectional study
Source: PLoS One. 2020 Jun 19;15(6):e0234632. doi: 10.1371/journal.pone.0234632 (PMC7304580; doi:10.1371/journal.pone.0234632)
Supplement: S1 Table — Going left to right, the columns contain information on: the extent of dilution, concentrations of N-Brev or Brev-A in diluted serum samples, percent recovery of N-Brev or Brev-A in serum diluted from 1:2 to 1:5. Optimal dilution for each assay is indicated by black square. (DOCX) [file pone.0234632.s003.docx]

| Dilution recovery in N-Brev ELISA | | | | | | |
| --- | --- | --- | --- | --- | --- | --- |
| Sample name | Dilution of serum | Measured serum (ng/mL) | Recovery % from 1:2 | Recovery % from 1:3 | Recovery % from 1:4 | Recovery % from 1:5 |
| 1 | 1:2 | 5.6 |  |  |  |  |
|  | 1:3 | 4.5 | 120 |  |  |  |
|  | 1:4 | 3.5 | 125 | 105 |  |  |
|  | 1:5 | 3.0 | 133 | 111 | 106 |  |
|  | 1:6 | 2.7 | 141 | 118 | 113 | 106 |
| 2 | 1:2 | 7.9 |  |  |  |  |
|  | 1:3 | 6.6 | 125 |  |  |  |
|  | 1:4 | 5.7 | 144 | 115 |  |  |
|  | 1:5 | 5.3 | 168 | 134 | 116 |  |
|  | 1:6 | 4.7 | 179 | 143 | 124 | 107 |
| 3 | 1:2 | 6.8 |  |  |  |  |
|  | 1:3 | 5.3 | 116 |  |  |  |
|  | 1:4 | 4.5 | 132 | 114 |  |  |
|  | 1:5 | 3.7 | 136 | 118 | 104 |  |
|  | 1:6 | 3.6 | 158 | 137 | 120 | 116 |
| 4 | 1:2 | 6.9 |  |  |  |  |
|  | 1:3 | 5.7 | 124 |  |  |  |
|  | 1:4 | 4.5 | 132 | 107 |  |  |
|  | 1:5 | 4.2 | 152 | 123 | 115 |  |
|  | 1:6 | 3.9 | 169 | 137 | 128 | 111 |
| Mean |  |  | **141** | **122** | **116** | **110** |
| Dilution recovery in Brev-A ELISA | | | | | | |
| 1 | 1:2 | 2.7 |  |  |  |  |
|  | 1:3 | 1.8 | 99 |  |  |  |
|  | 1:4 | 1.3 | 94 | 95 |  |  |
|  | 1:5 | 1.3 | 119 | 121 | 127 |  |
|  | 1:6 | 1.1 | 118 | 120 | 126 | 99 |
| 2 | 1:2 | 3.7 |  |  |  |  |
|  | 1:3 | 2.7 | 110 |  |  |  |
|  | 1:4 | 1.9 | 105 | 96 |  |  |
|  | 1:5 | 1.8 | 126 | 115 | 120 |  |
|  | 1:6 | 1.6 | 129 | 117 | 123 | 102 |
| 3 | 1:2 | 4.7 |  |  |  |  |
|  | 1:3 | 2.9 | 94 |  |  |  |
|  | 1:4 | 2.5 | 106 | 112 |  |  |
|  | 1:5 | 2.1 | 113 | 120 | 107 |  |
|  | 1:6 | 1.7 | 108 | 115 | 102 | 96 |
| 4 | 1:2 | 7.6 |  |  |  |  |
|  | 1:3 | 5.4 | 108 |  |  |  |
|  | 1:4 | 4.2 | 110 | 102 |  |  |
|  | 1:5 | 3.2 | 107 | 99 | 97 |  |
|  | 1:6 | 2.7 | 106 | 98 | 96 | 99 |
| Mean |  |  | **110** | **110** | **112** | **99** |
