# Supplementary material for: The CNS-specific proteoglycan, brevican, and its ADAMTS4-cleaved fragment show differential serological levels in Alzheimer’s disease, other types of dementia and non-demented controls: A cross-sectional study
Source: PLoS One. 2020 Jun 19;15(6):e0234632. doi: 10.1371/journal.pone.0234632 (PMC7304580; doi:10.1371/journal.pone.0234632)
Supplement: S2 Table — Going left to right, the columns contain information on: measured concentrations of N-Brev and Brev-A in serum samples, measured concentration of cleaved or full length rh-brevican for spiking, expected concentration of spiked serum samples, measured concentration of spiked serum samples, percent recovery of cleaved or full length rh-brevican and mean percent recovery in all samples. (DOCX) [file pone.0234632.s004.docx]

| Recovery of full-length rh-brevican - N-Brev | | | | | |  |
| --- | --- | --- | --- | --- | --- | --- |
| Sample name | Serum (ng/mL) | Spike (ng/mL) | Expected spiked serum (ng/mL) | Measured spiked serum (ng/mL) | Recovery % | Mean recovery % |
| 1 | 2.9 | 37.6 | 40.5 | 39.6 | 98 | 97 |
| 2 | 3.9 | 37.6 | 41.4 | 39.8 | 96 |  |
| 1 | 2.9 | 9.4 | 12.3 | 12.2 | 99 | 100 |
| 2 | 3.9 | 9.4 | 13.2 | 13.3 | 101 |  |
| 1 | 2.9 | 2.1 | 5.0 | 4.6 | 92 | 95 |
| 2 | 3.9 | 2.1 | 6.0 | 5.8 | 97 |  |
| 1 | 2.9 | 0.6 | 3.5 | 3.8 | 108 | 100 |
| 2 | 3.9 | 0.6 | 4.5 | 4.1 | 92 |  |
| 1 | 2.9 | 0.4 | 3.3 | 3.6 | 108 | 101 |
| 2 | 3.9 | 0.4 | 4.3 | 3.9 | 93 |  |
| Mean |  |  |  |  |  | **99** |
| Recovery of ADAMTS4-cleaved rh-brevican - Brev-A | | | | | | |
| 1 | 0.9 | 1.6 | 2.5 | 2.7 | 109 | 102 |
| 2 | 3.5 | 1.6 | 5.1 | 5.0 | 98 |  |
| 3 | 3.1 | 1.6 | 4.7 | 4.6 | 97 |  |
| 4 | 0.6 | 1.6 | 2.2 | 2.3 | 104 |  |
| 1 | 0.9 | 0.5 | 1.4 | 1.5 | 104 | 98 |
| 2 | 3.5 | 0.5 | 4.0 | 3.7 | 91 |  |
| 3 | 3.1 | 0.5 | 3.7 | 3.4 | 93 |  |
| 4 | 0.6 | 0.5 | 1.1 | 1.2 | 105 |  |
| 1 | 0.9 | 0.2 | 1.1 | 1.1 | 106 | 101 |
| 2 | 3.5 | 0.2 | 3.7 | 3.4 | 92 |  |
| 3 | 3.1 | 0.2 | 3.3 | 3.1 | 94 |  |
| 4 | 0.6 | 0.2 | 0.7 | 0.8 | 113 |  |
| Mean |  |  |  |  |  | **105** |
