# Supplementary material for: The CNS-specific proteoglycan, brevican, and its ADAMTS4-cleaved fragment show differential serological levels in Alzheimer’s disease, other types of dementia and non-demented controls: A cross-sectional study
Source: PLoS One. 2020 Jun 19;15(6):e0234632. doi: 10.1371/journal.pone.0234632 (PMC7304580; doi:10.1371/journal.pone.0234632)
Supplement: S3 Table — Listed are the Spearman’s rho correlation coefficients (r) with the 95% confidence interval. Aβ, amyloid-β; T-tau, total tau; P-tau, phosphorylated tau; n, number of patients. (DOCX) [file pone.0234632.s005.docx]

|  | **N-Brev** | |
| --- | --- | --- |
|  | R (95% CI) | p-value |
| Aβ (n = 114) | 0.09 (-0.09-0.27) | 0.32 |
| T-tau (n = 119) | 0.15 (-0.03-0.32) | 0.09 |
| P-tau (n = 120) | 0.20 (0.02-0.37) | 0.02 |
