# Supplementary material for: The CNS-specific proteoglycan, brevican, and its ADAMTS4-cleaved fragment show differential serological levels in Alzheimer’s disease, other types of dementia and non-demented controls: A cross-sectional study
Source: PLoS One. 2020 Jun 19;15(6):e0234632. doi: 10.1371/journal.pone.0234632 (PMC7304580; doi:10.1371/journal.pone.0234632)
Supplement: S4 Table — For ROC curve analysis performed on serum levels of N-Brev and Brev-A, reported are the AUC, optimal cut-off values calculated by the Youden index and the corresponding measures of sensitivity and specificity for each comparison. (DOCX) [file pone.0234632.s006.docx]

| Biomarker | Groups | AUC [95% CI] | Cut-off (ng/mL) | Sensitivity  (%) | Specificity  (%) | p-value |
| --- | --- | --- | --- | --- | --- | --- |
| N-Brev |  |  |  |  |  |  |
|  | OD, AD | 0.66 [0.53-0.77] | 5.9 | 59 | 82 | 0.02 |
|  | OD, NDCs | 0.65 [0.54-0.75] | 7.8 | 73 | 63 | 0.02 |
|  | NDCs, AD | 0.54 [0.42-0.66] | 11.0 | 82 | 37 | 0.56 |
| Brev-A |  |  |  |  |  |  |
|  | OD, AD | 0.61 [0.49-0.73] | 0.8 | 37 | 89 | 0.04 |
|  | OD, NDCs | 0.62 [0.51-0.72] | 0.7 | 39 | 90 | 0.02 |
|  | NDCs, AD | 0.50 [0.36-0.62] | 2.1 | 7 | 100 | 0.99 |
